# Imaging findings in the temporomandibular joint complex following radiation therapy for head and neck cancer: a review

**DOI:** 10.1186/s13244-026-02319-z

**Published:** 2026-06-13

**Authors:** Andrew Nalley, Madeline Yon, Kuo Feng Hung, Andy Wai Kan Yeung, Gang Li, Yiu Yan Leung, Tiffany Y. So, Ho Sang Leung, Qi Yong H. Ai

**Affiliations:** 1 Oral and Maxillofacial Radiology, Applied Oral Sciences and Community Dental Care, Faculty of Dentistry, The University of Hong Kong, Hong Kong, China; 2 Centre for Oral Clinical Research, Institute of Dentistry, Queen Mary University of London, London, United Kingdom; 3 Department of Oral and Maxillofacial Radiology, Peking University School and Hospital of Stomatology, Beijing, China; 4 Division of Oral and Maxillofacial Surgery, Faculty of Dentistry, The University of Hong Kong, Hong Kong, China; 5 Department of Imaging and Interventional Radiology, The Chinese University of Hong Kong, Prince of Wales Hospital, Hong Kong, China

**Keywords:** TMJ, Trismus, Radiotherapy, Radiology, Head and neck cancer

## Abstract

**Objectives:**

This study aimed to identify and synthesize imaging findings in the temporomandibular joint (TMJ) complex following radiation therapy (RT) for head and neck cancer (HNC).

**Materials and methods:**

A systematic search of five databases retrieved studies reporting post-RT imaging of TMJ structures. Extracted data included patient demographics, RT dose and technique, imaging modality, imaging changes and clinical findings. Risk of bias was assessed using ROBINS-I V2 (Risk of bias in non-randomized studies—of interventions, version 2).

**Results:**

Eleven retrospective studies (282 patients; mean age 52.7 years; RT dose 30–110 Gy) were included. Post-RT analysis was evaluated by imaging modalities of MRI (7 studies), CT (4), US (1), and panoramic images (1). The most commonly described structural changes in the muscles of mastication included MRI signal abnormalities (43 patients), atrophy (20 patients) and hypertrophy (20 patients), while condylar changes included erosion (11 patients), surface irregularity (12 patients) or sclerosis (6 patients). Trismus was identified in 126 patients (44.7%). No study described changes to the glenoid fossa or articular eminence or direct osteoradionecrosis (ORN) of TMJ structures. All studies exhibited moderate to critical risk of bias, limiting the certainty of associations.

**Conclusions:**

RT can induce structural changes across TMJ components detectable on imaging, which may contribute to trismus and functional impairment. Current evidence is limited, heterogeneous and retrospective, underscoring the need for prospective studies with standardized imaging and clinical correlation.

**Key Points:**

- Radiation therapy for head and neck cancer can affect jaw structures. Imaging shows changes in muscles and joints that are involved with chewing and mouth opening.
- These changes may lead to trismus (difficulty opening the mouth). Nearly half of patients in reviewed studies experienced this condition, impacting daily life.
- Current evidence is limited and inconsistent. Prospective studies with standardized imaging approaches are needed to further clarify radiation-related TMJ changes.

**Graphical Abstract:**

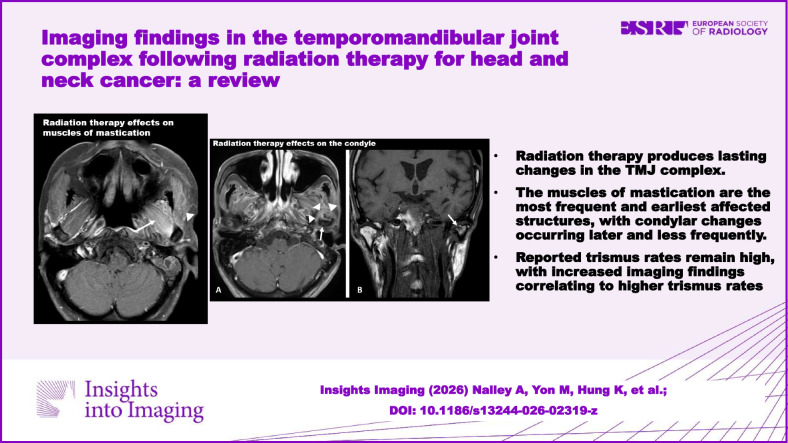

## Introduction

Head and neck cancer (HNC) is common worldwide, with the most common histological type being squamous cell carcinoma (SCC). HNC exhibits wide variation in incidence across anatomical sites, geographic regions, and patient demographics. It most commonly arises in the oral cavity and oropharynx, while cases involving the nasopharynx (NP) and larynx are becoming increasingly prevalent [1–7].

Treatment for HNC can be complex, depending on the location and stage of disease, and radiation therapy (RT) is frequently used as a standalone option in early cases or as an adjunct to surgery in more advanced cases [8, 9]. Up to 80% of HNC cases require RT as part of treatment [10, 11]. Advances in RT techniques, such as intensity-modulated radiation therapy (IMRT), have improved dose conformity and reduced exposure to adjacent structures [12–14]. However, post-radiation complications can manifest at varying intervals and occur during both early and late phases of the treatment course [15–20].

Trismus, defined as mouth opening of ≤ 35 mm [21], affects up to 60% of patients following RT and may develop rapidly after treatment, with gradual progression over subsequent years [22–25]. This complication significantly impacts daily function and quality of life [22, 26–31]. The structures that are implicated in radiation-induced trismus have been the temporomandibular joint (TMJ), masseter muscles and pterygoid muscles [32–35], with fibrosis of the muscles identified as the most common cause [36–38]. In addition to limited mouth opening, RT has been found to increase the incidence of other symptoms consistent with temporomandibular disorder (TMD), such as stiffness, fatigue, inflammation, and pain [37, 39–42].

Previous reviews have evaluated the prevalence of trismus in HNC patients treated with RT [24, 28, 43–46], the potential risk factors for developing trismus [47, 48], and the comparison of different treatment options [26, 49–52]. However, few have systematically addressed all imaging-detected changes in all structures of the TMJ complex after RT. Only one review described the MRI appearance of the muscles of mastication after RT to the head and neck region [53]. Therefore, this review synthesizes imaging-based evidence of RT-induced changes in TMJ structures as seen through different imaging modalities and their potential clinical implications.

## Materials and methods

This review was reported following the Preferred Reporting Items for Systematic Reviews and Meta-Analyses (PRISMA) [54] guideline. The study protocol was registered with the International Prospective Register of Systematic Reviews, PROSPERO (registration code: CRD420251110689).

### Research question

In order to evaluate the full findings of the TMJ region, for this review, the TMJ complex was defined to include the osseous components (Condyle, Glenoid Fossa, Articular Eminence) and soft tissue components (Articular Disc, Joint Capsule, Muscles of Mastication, namely the Temporalis, Masseter, Medial and Lateral Pterygoid) that are directly involved with the form and function of the joint.

A clinical question was developed following the PICO format (Table 1), with the full statement as follows: In patients who have received radiation treatment involving the TMJ complex (P), how does radiation therapy (I), compared to before radiation treatment, no radiation or different radiation protocols (C), affect radiological pathologic findings in the structures of the temporomandibular joint (O).

**Table 1:** The PICO statement

Patient	Intervention	Comparison	Outcome
Patients who have undergone radiation therapy, regardless of patient’s age, indication for radiation treatment or the time since treatment	Radiation therapy targeting the head and neck regions, specifically with the structures of the TMJ complex included in the primary field	Patients who have not received radiation therapy, received different radiation protocols, or the same patient’s images prior to radiation therapy treatment	Radiological pathologic findings in the temporomandibular joints, such as osteoarthritic changes, joint space narrowing, condylar resorption, sclerosis, fibrosis, scarring or other structural alterations observed on imaging modalities like MRI, CT, ultrasound or panoramic radiographs

The inclusion criteria for studies were:

1. Original articles published in English;
2. Studies that included patients who had received RT to the head and neck region, regardless of patient’s age, indication for treatment or type of RT utilized;
3. Studies that included patients where the primary field of RT included the structures of the TMJ complex (Condyle, Glenoid Fossa, Articular Eminence, Articular Disc, Joint Capsule, Muscles of Mastication);
4. Retrospective or Prospective studies that included imaging evaluation of the TMJ complex by any imaging modality at any time frame after RT, regardless of clinical findings.

While the exclusion criteria for studies were as follows:

1. Full text is not available or accessible;
2. Review articles, letters to the editor, abstract presentations or conference posters;
3. Studies where the patient’s primary or metastatic disease processes or surgical interventions included the TMJ complex and thus interfered with imaging evaluation;
4. Studies that only included clinical evaluation of Trismus or TMD;
5. Animal or in vitro studies.

### Search strategy and selection process

A search strategy was designed to evaluate three main components for inclusion in the review. The strategy attempted to identify studies where (1) patients had received RT treatment previously for any indication and that included the structures of the TMJ complex (condyle, articular disc, articular component of the temporal bone, or muscles of mastication) in the primary field; (2) imaging analysis by any modality was carried out at any time after RT, and (3) analyzed and described the findings to the TMJ complex structures as caused by RT as seen on imaging. The search strategy keywords developed were:

(((“Radiation Therapy”) OR (“RT”) OR (“Radiotherapy”) OR (“Head and Neck Radiation”) OR (“Radiation Treatment”) OR (“Intensity Modulated”) OR (“Radiation Oncology”)) AND ((“Magnetic Resonance Imaging”) OR (“MRI”) OR (“Computed Tomography”) OR (“CT”) OR (“Ultrasound”) OR (“US”) OR (“Imaging”) OR (“Signal Alterations”) OR (“Enhancement”)) AND ((“TMJ”) OR (“Temporomandibular”) OR (“Temporomandibular Joint”) OR (“DJD”) OR (“Degenerative Joint Disease”) OR (“Lateral Pterygoid”) OR (“Medial Pterygoid”) OR (“Temporalis”) OR (“Masseter”) OR (“Articular Disc”) OR (“Disc Displacement”) OR (“Internal Derangement”) OR (“Glenoid Fossa”) OR (“Articular Eminence”)))

On July 23, 2025, the search keywords were entered into 5 electronic databases: PubMed, Scopus, MEDLINE, Embase (Ovid) and Web of Science. There were no restrictions in the search strategy for terms or publication period. The developed search terms identified a total of 1875 references. The records were collected into a reference manager (Rayyan™ [55]). The records were screened for duplicates identified by the software. The titles and abstracts of all records after duplicate removal were evaluated by two reviewers (A.N., M.Y.). Relevant studies were selected for full-text analysis. Studies that were able to be retrieved were evaluated by both reviewers. An additional manual search was completed, and the references of the selected articles were screened for more studies that complied with the inclusion criteria.

### Data extraction

Two independent authors (A.N., M.Y.) reviewed the included studies and extracted relevant information. The data items retrieved were as follows:

1. Authors;
2. Publication year;
3. Type of study;
4. Number of patients;
5. Characteristics of patients (age, gender);
6. Tumor type and location;
7. Type of RT and dose;
8. If concurrent chemotherapy treatment was given;
9. The field of radiation treatment;
10. The time between the RT and the imaging analysis;
11. The imaging modalities utilized;
12. The imaging findings of the TMJ complex;
13. The presence of RT-induced trismus.

The primary outcome of interest was any imaging-detectable structural or morphological alteration of the TMJ complex after RT. Imaging findings considered relevant included:

- Bone changes: e.g., cortical erosion, sclerosis, trabecular rarefaction, or condylar deformity.
- Muscle changes: e.g., atrophy, fibrosis, enhancement or signal alterations on MRI.
- ORN: areas of exposed or necrotic bone with non-healing characteristics, often accompanied by fragmentation or sequestration. Although ORN overlaps with bone changes, it was treated as a distinct category when explicitly diagnosed or described by authors.

### Risk of bias

A risk of bias assessment of included studies was developed using the ROBINS-I V2 (risk of bias in non-randomized studies—of interventions, version 2) [56] tool to evaluate. Studies were rated as having low, moderate, serious or critical risk of bias in 7 categories. These domains included risks due to: confounding, classification of interventions, selection of participants into the study, deviations from intended interventions, missing data, measurement of the outcome, and selection of the reported result. According to the criteria identified in the ROBINS-I V2 tool, the overall risk of bias was determined by the category with the highest risk. Reporting bias was evaluated by whether outcomes reported in each study were consistent with those prespecified in study protocols or trial registries when available. For studies without protocols, we examined discrepancies between methods and results to identify selective outcome reporting. Publication bias was planned to be assessed using funnel plots if ≥ 10 studies were included in any quantitative synthesis.

## Results

### Article identification

**Fig. 1:**
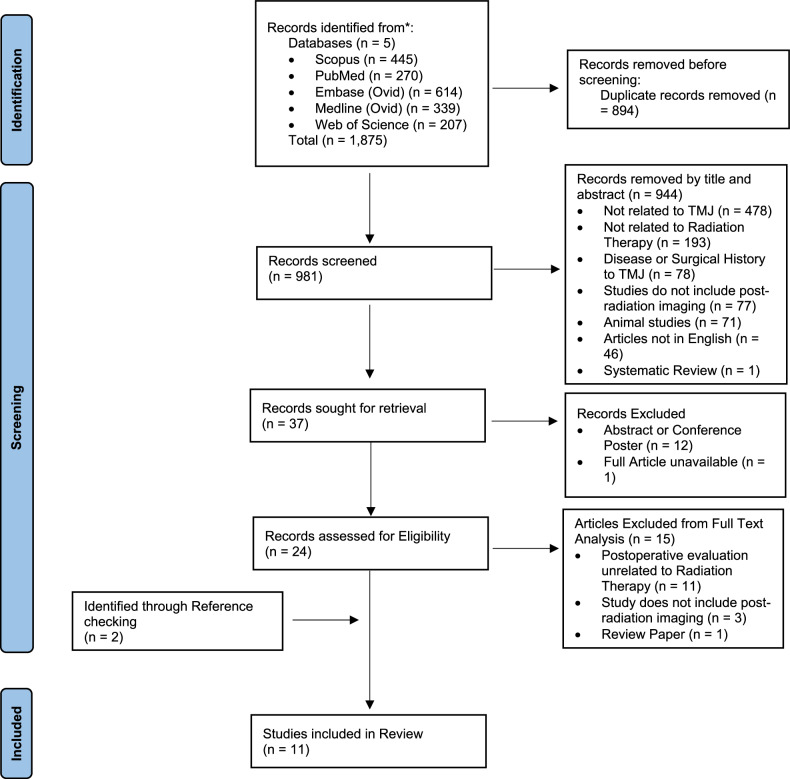
PRISMA flowchart of article selection process

Out of the 1875 articles, 894 duplicates were removed, leaving 981 articles for review. After title and abstract evaluation, full text was attempted to be retrieved for 37 articles. Out of these, 12 were conference posters or abstract submissions and one full text was not found. 24 articles underwent full-text review. During this full review, 11 articles did not include an analysis relevant to the effects of RT, 3 articles did not include evaluation by post-radiation imaging, and one article was a review paper. There were two additional articles included during this process by reference checking. A final total of 11 articles was included in this systematic review (Fig. 1).

All of the identified studies were retrospective in design, with four case reports or case series included (number of patients = 1, 1, 3 and 5, respectively). The 11 included studies ranged in time frame from 1996 to 2021.

Details of the following data from 11 eligible articles are shown in Table 2: numbers of patients, patient characteristics, cancer locations, RT techniques, RT field, concurrent chemotherapy (yes/no), time period between RT and post-RT imaging, post-RT imaging findings, and post-RT trismus (yes/no).

**Table 2:** Summary of data extracted from studies

Authors	Type of study	Number of patients	Patient demographics		Tumor type and location	Radiation therapy and dose	Radiation field	Concurrent chemotherapy	Post-RT time to imaging	Imaging modalities	TMJ complex findings	Trismus patients
			Age	Sex								
Pajari et al [62]	Case report	1	9	F	**Type**: RMS **Location**: Auricular with intracranial extension	**Conventional** 50 Gy	Primary site	Yes	1.5 years	OPG MRI	**MM:** Atrophy, Fibrosis, Higher electrical activity **Condyle**: Flattened head	1
Chong et al [61]	Review of patients with mandible ORN	5	•Avg: 54 •Range: 17–74	4 M 1 F	**Type**: 2 SCC, 1 ADC, 1 MEC, 1 OCC **Location**s: 1 OP, 1 NP, 3 SG	**Conventional** Total dose 60 to 69 Gy	Primary field included mandible	Not specified	Radiation to ORN diagnosis: 1 to 8 years	5 pts CT 3 pts MRI	**MM/LP:** 4/5 Enhancement and mass-like thickening on CT; 3/3 had signal abnormality on MRI	3
Ariji et al [73]	Case report	1	57	M	**Type**: Undifferentiated carcinoma **Location**: NP	**Conventional** **Primary site:** 75.8 Gy using 6MV x-ray **Neck:** 63.8 Gy using 6MV x-ray	Primary site and neck	Yes	**Two scans:** 5 months 8 months	MRI	**LP:** increased signal on T2WI **MM/MP**: slight enhancement on Gd-T1WI	0
Nichol et al [82]	CT imaging to quantify muscle atrophy	14	•Avg: 50.6 •Range: 29–88	5 M 9 F	**Type**: 4 MEC, 3 SCC, 1 AcCC, 3 ACC, 2 ADC, 1 LEC **Location**: 12 SG, 1 OP, 2 Other	**Conventional** **Total**: 50 to 66 Gy	Primary site and neck	No	Minimum of 1 year Median follow-up 2.9 years, Range 1 to 7.6 years	CT	**MM/LP**: Rate of atrophy 3.9% for MM and 2.3% for MP	0
Bhatia et al [58]	MRI findings in patients with severe trismus	35	•Avg: 51.2 •Range: 35–75	30 M 5 F	**Type**: 35 Undifferentiated carcinoma **Location**: 35 NP	Not specified	Primary site and neck	Not specified	Avg time: 6.7 years, Range 1.3 to 15.2 years	MRI	**Muscles:** 54% of pt signal abnormality. •3% had fibrotic scar tissue and atrophy. **Condyle**: 14% had collapsed and sclerotic condyle	35
Hsieh et al [67]	MRI-based scoring to predict trismus severity	22	•Avg: 50 •Range: 38–71	20 M 2 F	**Type**: 22 SCC **Location**: 22 OC	**IMRT** **High Risk**: 64–66 Gy. **Intermediate**: 60 Gy. **Low Risk**: 51–54 Gy	Primary site and neck	•Yes—20 patients •No—2 patients	**Three Scans:** 6 months, 12 months and 24 months	MRI	**Muscles:** All patients had signal abnormality; Trismus group higher abnormality 9% had atrophy **Condyles**: mentioned as risk factor for poor prognosis	17
Ahmed et al [60]	CT in patients with temporal bone ORN	20	•Avg: 61 •Range: 28–91	13 M 7 F	**Type**: 8 SCC, 1 BCC, 2 Melanoma, 4 Undifferentiated carcinoma, 5 Other **Location**: 9 SG, 4 NP, 2 OC, 5 Other	**IMRT**: 1 patient. Not specified: 19 patients •Avg Dose: 60 Gy •Range: 30 to 75.6 Gy	Primary site and neck	Not specified	Time to onset of ORN: Median 7 years, Range 2 to 22 years	CT	**Condyle**: 3 had condylar erosion	1
Mercado et al [63]	Case series of pediatric patients with condylar erosion	3	•Avg: 14 •Range: 10–17	2 M 1 F	**Type:** ES, RMS, Medullo-blastoma **Location:** 1 OC, 1 NP, 1 Other	**IMRT** •Avg 53.4 Gy •Range 50.4–55.8 Gy	Primary site and neck	Yes	1: 2 years 2: 4 years 3: 4 years	CT	**Condyle:** Erosion, Asymmetry, Joint space narrowing with retrognathia	1
Thor et al [74]	Case-control for SCC patients	20: 10 with Trismus; 10 controls without	•Avg: 57 •Range: 48–64	16 M 4 F	**Type:** 20 SCC **Location:** 18 OP, 2 NP	**IMRT** •Dose: 70 Gy	Primary site and neck	Not specified	•Within 1 year post RT •Avg Time 7 months	MRI	**Muscles:** Multiple involvement in development of trismus. Haralick texture of MP indicator of increased risk of trismus	10
Wu et al [59]	Retrospective cross-sectional study of NPC patients	114 pts	•Avg: 53	•75 M •39 F	**Type:** 114 Undifferentiated carcinoma **Location:** 114 NP	**Conventional:** 59patients **IMRT:** 55patients •Mean TMJ dose: 41.4 Gy •Range 41–45 Gy	Not specified	Not specified	•All pts 4+ years •Weighted average 14.9 years; median 15.5 years	US	**Muscles:** slightly more hypoechoic **Condyle:** Surface Irregularity = estimated prevalence ~12 pts based on 2 views; higher joint vascularity **Articular Disc:** Thinner in pts than control, thinner in Trismus than non-Trismus	39; 26 from Conventional and 13 from IMRT
Ma et al [57]	Patients with advanced ORN	47	•Median: 56 •Range: 28–71 •29 Male	29 M 18 F	**Type:** •21 Undifferentiated carcinoma •26 Others **Location:** •21 NP •7 OP •13 OC •6 Other	•Mean dose: 62 Gy •Range: 40–110 Gy •27 pts less than 66 Gy •20 66 Gy or more •12 pts received higher than 70 Gy	Not specified	14 (29.8%)	•Median follow-up time 27 months •Range 12 to 46 months	MRI	**Muscles:** 34% hypertrophy, •12.8% atrophy •53.2% edema •48.9% muscular fibrosis and infection	19
Total	•All studies were retrospective •2 Case report •2 Case series •2 Studies included control groups	282	•Avg (235): 52.7 Range: 9–91 •Median (47): 56	195 M 87 F (69.1%) (30.9%)	**Type:** •171 Undifferentiated carcinoma •55 SCC •56 Others **Location:** •179 NP •27 OP •37 OC •24 SG •15 Others	•Range: 30–110 Gy •Weighted Avg: 52.9 Gy •Median: 58 Gy		39 (13.8%)	•Weighted Avg: 8.13 years •Median: 6.7 years •Range: 5 months–22 years	**# Studies:** 1 OPG 6 MRI 4 CT 1 US		126 (44.7%)

*TMJ* temporomandibular joint, *ORN* osteoradionecrosis, *OPG* orthopantomogram, *CT* computed tomography, *US* ultrasound, *MRI* magnetic resonance imaging, *RT* radiation therapy, *IMRT* intensity-modulated radiation therapy, *Avg* average, *RMS* rhabdomyosarcoma, *SCC* squamous cell carcinoma, *MEC* mucoepidermoid carcinoma, *OCC* oncocytic carcinoma, *AcCC* acinic cell carcinoma, *ACC* adenoid cystic carcinoma, *ADC* adenocarcinoma, *LEC* lymphoepithelial carcinoma, *BCC* basal cell carcinoma, *ES* Ewing’s sarcoma, *NP* nasopharynx, *OP* oral pharynx, *OC* oral cavity, *SG* salivary gland, *pt* patient, *MM* masseter muscle, *LP* lateral pterygoid, *MP* medial pterygoid, *TM* temporalis muscle, *MID* maximum incisal distance

### Patient characteristics

**Fig. 2:**
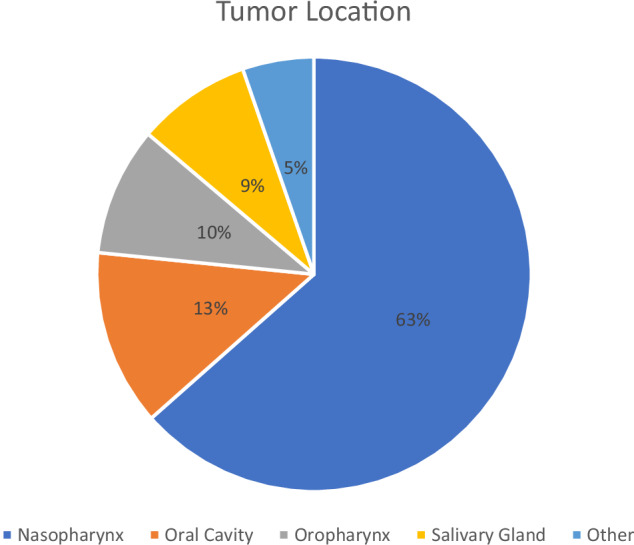
Primary tumor location

Collectively, there were a total of 282 patients between all studies who had received RT. Control patients who did not receive RT were not included in this total. The patient characteristics overall were 69.1% Male and 30.9% Female. The weighted average age from 10 of the studies was 52.7 years, while the study by Ma et al [57] only included the median age of 56 for their patients. The primary tumor location was 63.5% in the NP, 13.1% in the oral cavity, 9.6% in the oropharynx, 8.5% in the major salivary glands, and 5.3% in other locations (Fig. 2).

### Treatment details

**Fig. 3:**
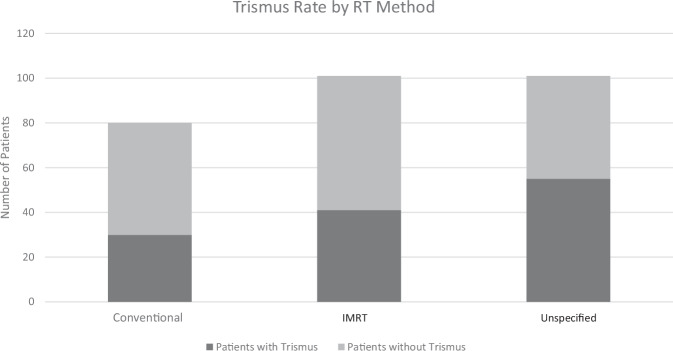
Number of patients who received RT by either conventional, IMRT or unspecified methods with the relative rates of trismus

For treatment, the radiation dose reported ranged from 30 Gy to 110 Gy. The study by Bhatia et al [58] did not include the radiation dose. The type of RT administered was solely conventional in 4 studies and solely IMRT in 3 studies. Bhatia et al and Ma et al did not indicate the type of RT. Wu et al [59] included 51.8% of patients who received conventional RT and 48.2% received IMRT, while Ahmed et al [60] included one example patient who received IMRT but did not clarify for the rest. In total, 80 patients received conventional RT, 101 received IMRT, while 101 patients were not specified for the delivery method of RT. Trismus occurred in 44.7% overall, with rates of 37.5%, 40.6% and 54.5% for conventional, IMRT and unspecified RT method, respectively. The number of patients who received these types of RT with the number of patients who developed trismus are displayed in Fig. 3.

Out of the 11 articles, 6 included clear descriptions of chemotherapy use, 4 did not specify chemotherapy use, while 1 shared incomplete reporting for the study population. From the studies that included this description, 39 patients had concurrent use of chemotherapy.

The time period between post-RT and post-RT imaging ranged from 5 months to 22 years, with a weighted average time of 8.1 years. The post-RT imaging was performed solely by MRI, CT or ultrasound in 5, 3, and 1 studies, respectively. Chong et al [61] reported on both CT and MRI, and the case report by Pajari et al [62] reported on a dental Panoramic and MRI images (Table 2).

### Findings by structure on post-RT imaging

In total, muscle alterations were reported in 9 studies; condylar changes in 5 studies, disc thinning in 1 study and joint capsule thickening in 1 study. The most commonly reported findings in the muscles of mastication included MRI signal abnormalities (*n* = 43 patients), atrophy (*n* = 20 patients), hypertrophy (*n* = 20 patients), edema (*n* = 25 patients), fibrosis and infection (*n* = 23 patients) and CT enhancement (*n* = 5 patients). The most commonly reported condylar findings included erosion (*n* = 11 patients), surface irregularity (*n* = 12 patients), sclerosis (*n* = 6 patients), and retrognathia (*n* = 2 patients). The soft tissue findings included joint capsule thickening (*n* = 2 patients) and articular disc thinning (identified in one study, number of patients not quantified). There were no direct findings of the glenoid fossa or articular eminence noted.

ORN was not identified originating from the condylar head or neck, or from the glenoid fossa or articular eminence. Two studies (Chong et al, Ma et al) identified changes occurring due to ORN originating in the mandible, while one study (Ahmed et al) identified changes due to ORN originating in the temporal bone.

**Fig. 4:**
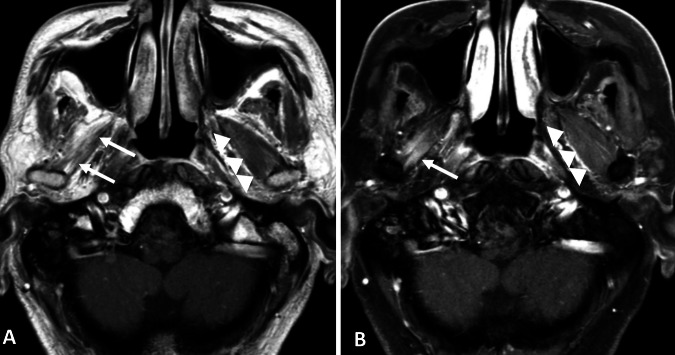
Post-treatment axial contrast-enhanced T1 (**A**) and fat-suppressed contrast-enhanced T1 (**B**) MRI images of a patient with nasopharyngeal carcinoma (NPC) treated with RT 4 years ago. The right lateral pterygoid muscle (arrow) displays diffuse enhancement signal and decreased size compared to the contralateral side (arrow heads), suggesting atrophy

**Fig. 5:**
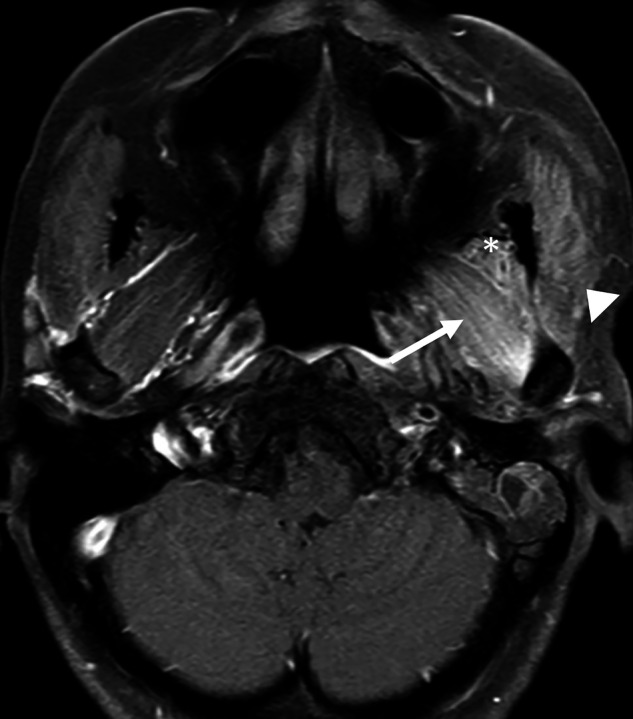
A post-treatment axial fat-suppressed contrast-enhanced T1W MRI image of a patient with left parotid myoepithelial carcinoma treated with RT 1 year ago. Radiation effects are seen in the left lateral pterygoid (white arrow), left temporalis (asterisk) and left masseter (white arrow head) muscles compared to the contralateral side

**Fig. 6:**
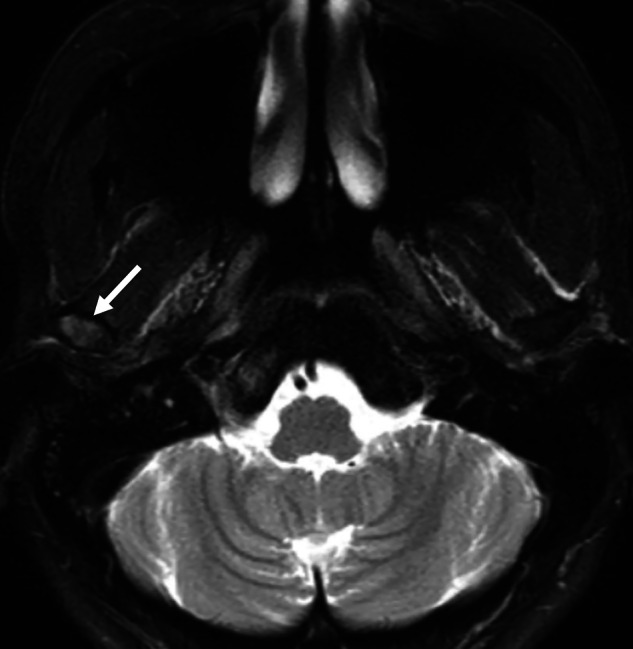
A post-treatment axial fat-suppressed T2W MRI image of a patient with right parotid adenoid cystic carcinoma treated with parotidectomy followed by adjuvant RT 2 years ago. There is an irregular high signal on the right mandibular condyle (white arrow), suggesting marrow inflammation or edema secondary to RT

**Fig. 7:**
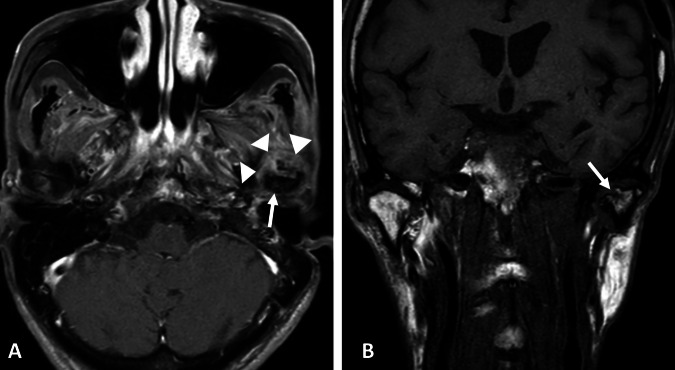
Post-treatment axial fat-suppressed contrast-enhanced T1W (**A**) and coronal contrast-free T1W (**B**) MRI of a patient with nasopharyngeal carcinoma (NPC) treated with RT over 20 years ago. The enhanced soft-tissue-like features (arrow heads) around the left mandibular condyle (arrows), the poorly marginal areas of the left mandibular condyle (arrows) and the decreased size of the left mandibular condyle (arrows) compared to the contralateral side could be sequelae of post-RT-induced inflammatory changes

Four examples of post-treatment MRIs of patients with head and neck cancer treated with RT that show post-RT changes in the TMJ complex are shown in Figs. 4–7. These MRI scans illustrate post-RT imaging appearances of the relevant structures.

When divided by RT delivery method, out of the patients who received conventional RT, 13 were identified with muscular atrophy, 2 with signal abnormality, 1 with increased electrical signal and 1 with condylar flattening. From the patients who received IMRT, 6 had muscular atrophy, 22 had signal abnormality, 3 had condylar erosion and 2 had condylar hypoplasia and mandibular retrognathia. The studies without a clear description of the RT method specified included 1 patient with atrophy, 16 with signal abnormality, 5 with collapsed and sclerotic condyles, and 2 with joint capsule thickening. The studies that evaluated effects from ORN included 5 patients who received conventional RT, 1 patient who received IMRT, and 66 who were unspecified. Out of these, 20 patients had muscular hypertrophy, 25 had edema, 23 had fibrosis, 3 had signal abnormalities, 5 displayed enhancement on CT, and 3 had condylar erosion.

**Fig. 8:**
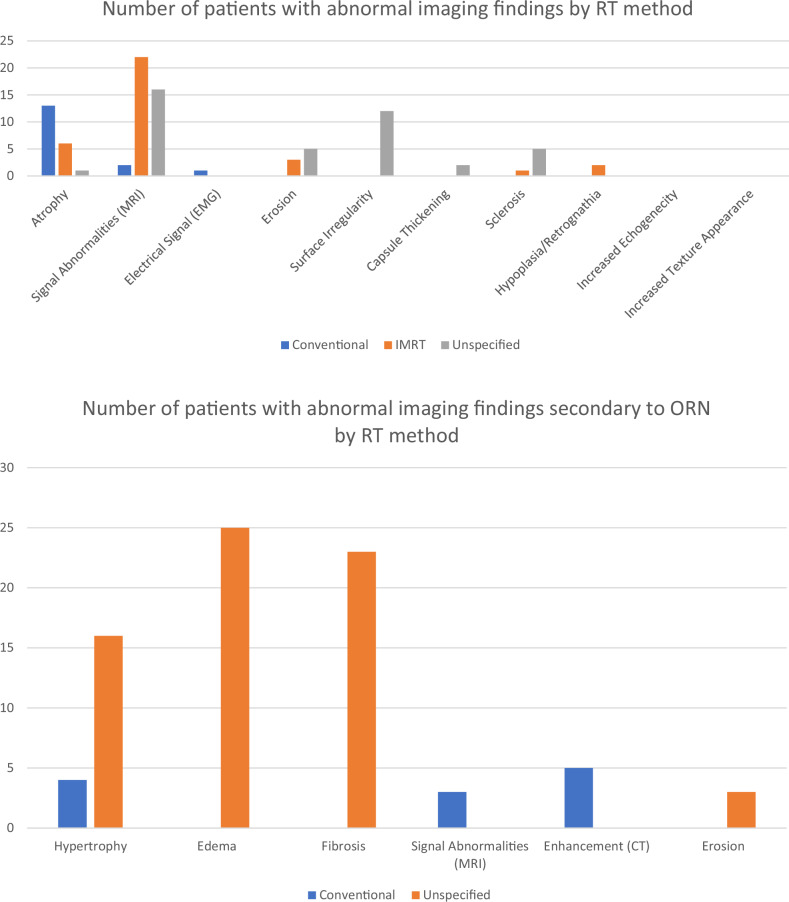
Summary of the number of patients with different imaging findings by RT method. Note that some studies identified the presence of findings without quantifying the specific number of patients

**Fig. 9:**
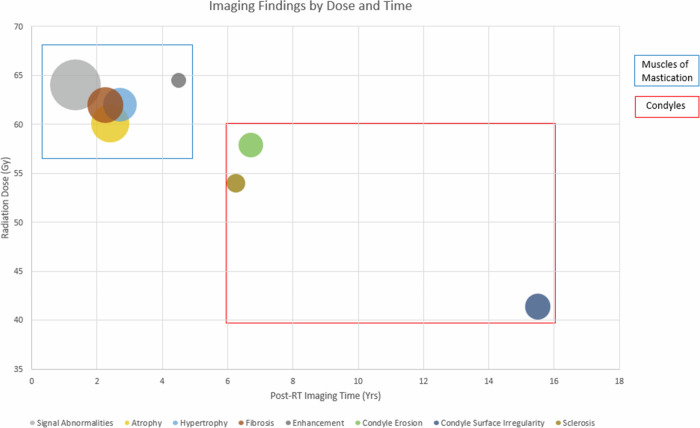
Bubble plots of the most commonly reported imaging findings from different studies by the weighted average for radiation dose and post-RT time until imaging analysis. The size of the bubble represents the number of patients described with the specified finding

The number of patients with the described imaging findings separated by RT type is displayed in Fig. 8. Bubble plots of the most common muscles of mastication and condyle findings relative to weighted averages of radiation dose received and imaging time until analysis can be found in Fig. 9.

### Risk of bias

Evaluating the 7 factors identified in the ROBINS-I V2 tool and classifying the overall risk of bias as the category with the highest risk of bias, 3 of the articles were rated with a critical risk, 4 were rated with a serious risk, and 4 were rated with a moderate risk. None of the articles achieved an overall low risk of bias. The results of the risk of bias are displayed in Table 3. Reporting bias assessment revealed that none of the included studies had publicly available protocols or trial registrations, limiting formal evaluation of selective outcome reporting. No major discrepancies between stated methods and reported results were identified. Publication bias assessment using funnel plots was not performed because quantitative synthesis was not conducted due to the high risk of bias and heterogeneity.

**Table 3:** ROBINS-I V2 risk of bias tool

	Bias due to confounding	Bias in classification of interventions	Bias in selection of participants into the study	Bias due to deviations from intended interventions	Bias due to missing data	Bias in measurement of the outcome	Bias in selection of the reported result	Overall risk of bias
Pajari et al, 1996	Critical	Moderate	Critical	Moderate	Serious	Serious	Critical	Critical
Chong et al, 2000	Serious	Moderate	Serious	Moderate	Moderate	Moderate	Serious	Serious
Ariji et al, 2002	Moderate	Low	Moderate	Low	Low	Low	Moderate	Moderate
Nichol et al, 2003	Moderate	Low	Moderate	Low	Low	Moderate	Moderate	Moderate
Bhatia et al, 2009	Serious	Moderate	Serious	Moderate	Moderate	Moderate	Serious	Serious
Hsieh et al, 2013	Moderate	Low	Moderate	Low	Low	Low	Moderate	Moderate
Ahmed et al, 2014	Critical	Moderate	Serious	Moderate	Moderate	Moderate	Serious	Critical
Mercado et al, 2014	Critical	Moderate	Critical	Moderate	Moderate	Serious	Serious	Critical
Thor et al, 2016	Moderate	Low	Low	Low	Low	Serious	Low	Serious
Wu et al, 2017	Moderate	Low	Moderate	Low	Low	Low	Moderate	Moderate
Ma et al, 2021	Moderate	Moderate	Serious	Moderate	Low	Serious	Moderate	Serious

## Discussion

RT induced changes in multiple TMJ structures across imaging modalities and time intervals.

### Imaging findings

Changes to the muscles of mastication were the most commonly and earliest reported finding across studies. The findings were mainly described on MRI but also observed on CT and US. The changes to the muscles ranged from minor to severe as seen on MRI, with greater signal or texture abnormalities correlating with higher trismus rates. The only two studies that did not discuss the findings of the muscles were evaluated solely by CT, with a focus on temporal bone ORN or a case series highlighting condylar erosion (Ahmed et al, Mercado et al [63]). Comparing the time frames between studies into early, middle and late stages, the changes to the muscles were described at all time points. The appearance of alterations in the muscles of mastication in the early, mid and late stages of evaluation in almost all studies likely indicates the sensitivity of the muscles to radiation-induced changes. Muscle abnormalities remained evident even with IMRT and the advantages of better RT field mapping. In fact, one study (Bhatia et al) specifically clarified that muscle signal alterations on MRI were nearly ubiquitous and therefore only reported markedly or striking changes.

Condylar changes, though less frequent, ranged from minor to severe. These included flattening, small osteophyte formation, and surface irregularity to severe sclerosis and erosive changes with collapsed height and morphology leading to retrognathia. Pediatric patients are more susceptible to radiation [64] and may be at increased risk of late RT effects [65, 66]. As such, the condyles may be more prone to damage from radiation in the pediatric population. In this review, both studies with pediatric patients focused on effects on the TMJ, ranging from minor to severe. However, this only included 4 patients in total. Five adult patients also exhibited severe condylar changes, suggesting this effect occurs across age groups. In addition, the study by Hsieh et al [67] did not describe changes to the joint but indicated it as a structure with a poor prognosis for trismus from increasing amounts of radiation dose.

There were no direct changes of the glenoid fossa or articular eminence described, such as erosion, sclerosis or ORN at these sites. This may indicate a decreased likelihood of radiation-induced changes to these structures. With an emphasis on selecting articles that specified the TMJ, other indications of RT to more posterior regions, such as the auditory canal, and their potential to affect these structures may not have been investigated in this study.

**Fig. 10:**
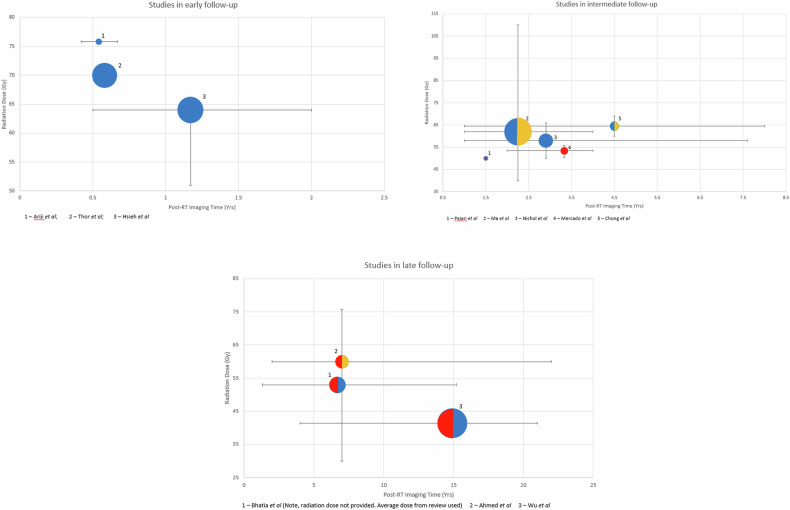
Bubble plots with error bars of all studies by average, weighted average or median time until imaging analysis post-RT. The studies were divided by quartile into early (<25%), intermediate (25–75%), and late (>75%)time to imaging follow-up. The error bars represent the range of radiation doses and time until imaging analysis from each study. The size of the bubble represents the number of patients included in the study. The color of the bubble represents the structure described with abnormal imaging findings—the muscles of mastication (blue), the condyle (red) and changes secondarily due to ORN (orange)

The time from completion of RT to imaging evaluation ranged widely. Alterations to the muscles were identified first, with changes seen as early as 5 months post-RT. Effects of the condyle were identified later, with the earliest changes seen at 18 months. To standardize reporting, studies were grouped by quartiles utilizing weighted average time from RT completion to imaging. The earliest quartile (≤ 1.25 years) was classified as ‘early,’ the interquartile range (1.26–5 years) as ‘middle’ and the highest quartile (> 5 years) as ‘late.’ This approach reflects relative timing across studies rather than fixed biological stages. Utilizing this framework, findings were observed across all stages, from the earliest months to decades after treatment, with the latest analysis occurring over 20 years post-RT. Overall, muscle changes appeared earlier than condylar changes, with all early-stage studies describing imaging findings in the muscles. The earliest condylar changes were reported at 1.5 years, and secondary changes related to ORN were observed as early as 1 year. The individual studies with their described findings are separated into quartiles by post-RT follow-up time and display the described imaging findings as a function of the range of radiation doses and times by bubble plots in Fig. 10.

Regarding ORN, there were no articles discovered that described ORN directly originating from the mandibular condyle or condylar neck, or from the glenoid fossa and articular eminence of the temporal bone. Three studies reported secondary TMJ complex involvement from ORN originating in the mandible or temporal bone. Temporal bone ORN has been described as having a localized form or a more severe, diffuse form which involves adjacent strufctures including the TMJ [68], which is the indication from Ahmed et al. Findings such as condylar erosion and muscular atrophy were described in these cases, similar to other studies. Other findings, such as muscular edema or hypertrophy, were only indicated in these studies and therefore may have been a result of the ORN inflammation rather than RT directly. While these did not directly describe RT’s effects on the TMJ, they were included to indicate a potential long term, indirect effect to these structures secondarily to the onset of ORN in other sites. These studies had high average radiation doses but also spanned the full range across all studies (35 to 110 Gy), highlighting the variability in dose associated with ORN development and its potential secondary effects on TMJ structures.

### Clinical indications

The overall rate of trismus of 44.7% is within other reported ranges of trismus development after RT [24, 69]. This included trismus rates of 37.5% for patients who received conventional RT, 40.6% from IMRT and 54.5% with unspecified RT method. It has been reported that IMRT decreases the rates of trismus development [70–72]; however, in this review, the patients who received IMRT had slightly higher trismus rates than the patients who received Conventional RT. Several factors limit the generalizability of trismus rates and may explain the slightly higher IMRT rates. The case study by Ariji et al [73] identified that the patient did not have trismus at the time of diagnosis, but this article did not identify post-RT clinical symptoms. The study by Ahmed et al included in the discussion one example patient with trismus but did not specify for the other 19 patients [60]. The study by Bhatia et al retrospectively identified all 35 of its patients as having trismus [58], while the study by Thor et al [74] retrospectively identified 10 patients with trismus compared to 10 patients without, introducing selection bias for the presence of trismus. These results indicate the continued importance of trismus even with the improvements from IMRT. Radiologists can contribute to prevention by integrating dose-volume data with early post-treatment imaging. Identifying patients at higher risk of trismus or detecting early imaging alteration enables timely referral for jaw mobilization therapy or supportive care services, potentially improving post-care outcomes [26, 49, 51, 52, 75].

Trismus following RT is multifactorial, with direct tissue injury and denervation atrophy contributing to its development; however, fibrosis is most frequently cited as the primary mechanism. This review identified nonspecific imaging-detected muscle alterations as the predominant and earliest reported findings, being able to be identified on MRI, CT and US images. In the combined cohort of 43 patients with muscular abnormalities detected on MRI, the majority (39 cases) were only described as nonspecific signal changes, with relatively few cases characterized in greater detail: three cases reported as T2 hyperintensity and one case described with T1 enhancement. Fibrosis was quantitatively reported in one study by Ma et al, which involved 23 patients who developed fibrotic changes in muscles adjacent to mandibular ORN. These imaging findings, which appeared as early as 5 months post-RT, support an early and targeted radiologic surveillance of the masticatory muscles. In addition to muscular involvement, additional alterations were noted in the TMJ complex, including the condyle, joint capsule and joint space. Because condylar alterations emerged later than muscular changes, longer-term surveillance is warranted—particularly in pediatric patients, whose condyles demonstrated greater susceptibility to growth disturbance and deformity.

These structures are functionally and anatomically interconnected, and changes in the muscles of mastication may influence the adjacent TMJ components [76]. Given that muscles exhibit the earliest radiation-induced changes, their potential impact on TMJ function and secondary morphological adaptations should be considered alongside direct RT damage. Consequently, functional assessment and continued long-term monitoring remain warranted.

ORN was not identified within the TMJ complex in our systematic review, suggesting a low likelihood for its direct involvement. Therefore, routine surveillance for ORN and complications such as osteomyelitis in these structures may be unnecessary. However, clinicians should remain vigilant for secondary effects arising from ORN in adjacent regions, such as the mandible or posterior temporal bone, as these can increase the risk of complications to all structures evaluated.

### Limitations

This review is limited by retrospective design, varieties in imaging protocols, and the absence of pre-treatment assessments. A meta-analysis was not completed because all studies had a moderate to critical risk of bias on ROBINS-I V2. Retrospective designs restricted control of confounding variables, and comparison groups varied widely: some studies compared to non-RT patients, others to contralateral sides or pre-treatment images, while most (6 studies) did not include a comparison. The studies selected the population based on the presence of ORN, NPC, SCC or trismus, introducing various levels of selection bias.

The described imaging findings experienced challenges in proper synthesis due to the heterogeneity of data and reporting. For purposes of comparison, the muscles of mastication and minor differences in imaging description were combined. In instances of insufficient clarity for the number of patients with a specific condition, the lowest number reported was used.

Reporting of chemotherapy was inconsistent—only 13.8% of patients had clear concurrent use, but 5 out of 11 studies did not provide treatment clarity. Chemotherapy combined with RT may have a higher incidence of trismus than RT alone [77], so this uncertainty limits attribution of findings solely to RT. Similarly, none of the studies addressed pre-existing TMD, which is a risk factor for increased risk of trismus [78]. TMD incidence is frequently reported to be 5–12%, but may be as high as 35% [79, 80]. On imaging analysis, TMD can cause imaging changes (e.g., condylar erosion, sclerosis, muscle alterations) [76, 79, 81] that overlap with post-RT findings.

Finally, variability in imaging interpretation introduced ambiguity. These methodological and descriptive inconsistencies, combined with heterogeneous imaging targets and modalities, challenge synthesis and prevent estimation of prevalence or causality.

## Conclusion

Head and neck cancer continues to affect a large number of patients worldwide and has a high indication for radiation therapy as part of the treatment regimen. Although the techniques of radiation therapy have improved and allowed for decreased radiation dose to adjacent structures, unintended structural effects, abnormalities and damage as seen on imaging may occur at multiple different sites related to TMJ form and function. A prospective, randomized controlled trial controlling various confounding variables has not been conducted, limiting the generalizability of these findings.

## Abbreviations

CT: Computed tomography
HNC: Head and neck cancer
IMRT: Intensity-modulated radiation therapy
MRI: Magnetic resonance imaging
NP: Nasopharynx
ORN: Osteoradionecrosis
RT: Radiation therapy
SCC: Squamous cell carcinoma
TMD: Temporomandibular joint disorder
TMJ: Temporomandibular joint
US: Ultrasound
